# BK_Ca_ Mediates Dysfunction in High Glucose Induced Mesangial Cell Injury via TGF-*β*1/Smad2/3 Signaling Pathways

**DOI:** 10.1155/2020/3260728

**Published:** 2020-04-29

**Authors:** Zhigui Wu, Wenxian Yin, Mengqi Sun, Yuankai Si, Xiaoxiao Wu, Meijuan Chen

**Affiliations:** ^1^Department of Pharmacy, Affiliated Hospital of Southwest Medical University, Luzhou 646000, Sichuan, China; ^2^Department of Pharmacy, Affiliated Hospital of Traditional Chinese Medicine Southwest Medical University, Luzhou 646000, Sichuan, China; ^3^Department of Pharmacy, Southwest Medical University, Luzhou 646000, Sichuan, China

## Abstract

**Objective:**

To explore the role and mechanism of BK_Ca_ in diabetic kidney disease.

**Methods:**

Rat mesangial cells (MCs) HBZY-1 were cultured with high glucose to simulate the high-glucose environment of diabetic kidney disease in vivo. The effects of large conductance calcium-activated potassium channel (BK_Ca_) on proliferation, migration, and apoptosis of HBZY-1 cells were observed. The contents of transforming growth factor beta 1 (TGF-*β*1), Smad2/3, collagen IV (Col IV), and fibronectin (FN) in the extracellular matrix were also observed.

**Results:**

High glucose significantly damaged HBZY-1 cells, which enhanced the ability of cell proliferation, migration, and apoptosis, and increased the secretion of Col IV and FN. Inhibition of BK_Ca_ and TGF-*β*1/Smad2/3 signaling pathways can inhibit the proliferation, migration, and apoptosis of HBZY-1 cells and suppress the secretion of Col IV and FN. The effect of excitation is the opposite.

**Conclusions:**

BK_Ca_ regulates mesangial cell proliferation, migration, apoptosis, and secretion of Col IV and FN and is associated with TGF-*β*1/Smad2/3 signaling pathway.

## 1. Introduction

The diagnosis of type 2 diabetes mellitus (T2DM), the majority of diabetes mellitus (DM), is often accompanied by chronic microvascular or macrovascular complications with high economic and social costs, among which are diabetic kidney disease (DKD), retinopathy, peripheral blood vessels, and coronary atherosclerosis. About 30% of DM patients suffer from DKD, the leading cause of end-stage kidney disease (ESKD) and even premature death [1]. Glomerulus is currently regarded as the main site of lesions of DKD with the main pathological features being diffuse mesangial matrix dilatation, exudative lesions and segmental nodular sclerosis in the glomeruli [2], accumulation of the extracellular matrix (ECM) in the glomerulus and tubulointerstitial septal compartment [3], and thickening and transparency of the intrarenal vascular system [4]. Glomerular mesangial cells (MCs) are involved in many physiological activities, such as the production of growth factors, the formation of glomerular mesangial matrix as the structural support of capillaries, and the regulation of glomerular hemodynamics by contractile properties [5]. When the glomerulus is damaged, MCs often change their phenotype to myofibroblasts expressing *α*-smooth muscle actin or interstitial collagen besides normal matrix components, which is a key link of glomerulosclerosis [6]. Despite many strategies proven effective, including control of blood sugar and blood pressure and inhibition of the renin-angiotensin-aldosterone system (RAAS), the number of DM patients who eventually develop DKD is still large [7]. Therefore, it is still vital to find new therapeutic targets for preventing and delaying the progress of DKD.

BK_Ca_ is considered to be a key participant in many physiological functions, including regulating neuronal discharge [8], smoothing muscle tension [9], promoting endocrine cell secretion [10], cell proliferation and migration [11], etc. It also participates in a series of diseases, such as hypertension, epilepsy, cancer, and so on [12]. Recently, BK channel subtypes were also found in glomerular podocytes and mesangial cells [6, 13]. Our previous studies found that BK_Ca_ channel expression was downregulated and the current density of the BK_Ca_ channel was downregulated in diabetic coronary artery smooth muscle cells [14]. TGF-*β*1 is a key regulator of ECM synthesis and degradation in diabetic nephropathy [15], which promotes renal fibrosis by upregulating the gene encoding ECM proteins and enhancing the production of ECM degrading enzyme inhibitors to inhibit its degradation [16]. TGF-*β*1 first binds to the membrane TGF-*β*II receptor and complexes are transphosphorylated to reactivate type I receptor, and then it activates Smad signaling pathway and regulates the transcription of TGF-*β*1 target genes, such as Col IV and FN, through phosphorylation of Smad 2/3 and translocation into the nucleus [17, 18].

At present, no research has focused on the relationship between BK_Ca_ and MCs. However, BK_Ca_ participates in cell proliferation and migration and can regulate the secretion of endocrine cells; MCs can secrete mesangial matrix, transform the phenotype into fibroblasts under stress, and participate in glomerulosclerosis. Due to the similar function between the two, it is worth further exploring their internal relationship.

In this study, HBZY-1 cells were cultured with high glucose to establish the DKD cell model. The effects of inhibiting or activating BK_Ca_ on the proliferation, migration, and apoptosis of HBZY-1 cells, as well as the changes of TGF-*β*1, Smad2/3, and ECM (Col IV, FN) were observed to explore the role of BK_Ca_ in DKD and its mechanism, so as to provide new ideas for the clinical treatment and drug development of DKD.

## 2. Materials and Methods

### 2.1. Morphological Characteristics and Experimental Grouping of HBZY-1 Cells

The morphological characteristics of HBZY-1 cells (purchased from the China Center for Type Culture Collection) under high- and low-sugar conditions (cultured with 10% fetal bovine serum, 100 U/ml penicillin, and 100 mg/ml streptomycin at 37°C in 95% air and 5% CO_2_) were observed under a microscope, and the cells at logarithmic growth stage were selected for experiment. The cells were transfected with BK_Ca_-siRNA (the details of RNA interference are available in the supplementary materials). The mesangial cells were intervened with BKCa agonist NS11021 [19] or inhibitor tetrandrine (Tet) [20]and TGF-*β*1 or inhibitor SB431542 [21].

The experiment was divided into 9 groups: low-glucose control group (NG, 5.5 mmol/L glucose), high-glucose control group (HG, 24.5 mmol/L glucose), BK_Ca_-siRNA group (HG + BK_Ca_ gene expression inhibition), Tet group (HG + Tet 10 *μ*M), NS11021 group (HG + NS11021 10 *μ*M), TGF-*β*1 group (HG + TGF-*β*12 ng/ml), SB431542 group (HG + SB431542 10 *μ*M), NS11021 + SB1442 group (HG + NS11021 10 *μ*M + SB431542 10 *μ*M), and Tet + TGF-*β*1 group (HG + Tet 10 *μ*M + TGF-*β*12 ng/ml).

### 2.2. Mesangial Cell Proliferation

MTT was used to measure the cell survival rate to calculate the proliferation. The cells were counted and the density was adjusted to 5 × 10^4^ and then inoculated into 96-well plates at 37°C in a 5% CO_2_ incubator for 24 hours. Then corresponding intervention drugs were added according to the experimental design; 5 ml MTT solution was added to each hole, and then the cells were cultured for 4 hours. The supernatant should be absorbed and discarded, and 150 *μ*L of DMSO was added to each well. They were shaken for 10 minutes. After crystallization was completely dissolved, the OD value (absorbance) at 490 nm wavelength of each pore was detected by using enzyme labeling instrument.

The cell survival rate was calculated by the following equation: (OD administration − OD blank)/(OD control − OD blank)*∗*100%.

### 2.3. HBZY-1 Cell Migration

Three horizontal lines were first drawn evenly in each hole back of the 6-well plate, and then HBZY-1 cells were cultured with a density of 12 × 10^4^ at 37°C in a 5% CO_2_ incubator for 24 hours. Then, 3 scratches were made in the cells evenly in each hole perpendicular to the previous horizontal line with the tip of the pipette and continually cultured for grouping intervention; photographs were taken at 0 and 48 hours, and the scratch area was calculated by image J software. Use ImageJ software to open the picture to be analyzed. Under the Process project, click Enhance Contrast, select Normalize, adjust the Saturated pixels to 0.3%, and then select Smooth and Find Edges under the Process project. After that, select Adjust-Threshold under the Image item, select Red, and adjust the threshold to 0−20. Finally, use the magic wand tool to select the black scratches and select Measure under the Analyze project to get the scratch area. Wound healing percentage = initial scratch area/scratch area at a point in time.

### 2.4. Hoechst Dyeing Experiment

HBZY-1 cells were cultured into 6-well plates at 8 × 10^4^ density at 37°C in a 5% CO_2_ incubator for 24 hours. The cells were intervened by adding drugs in groups after 48 hours, the supernatant was discarded, and 4% polyformaldehyde fixed solution and Hoechst 33258 staining solution were added to each hole, respectively. After discarding the staining solution, the cell staining was observed under an inverted fluorescence microscope and then photographed, recorded, and analyzed.

### 2.5. Annexin V-FITC/PI Double-Staining Experiment

HBZY-1 cells were planted into 6-well plates at 12 × 104 density. The cells were cultured at 37°C in a 5% CO_2_ incubator for 24 hours, then treated in groups, and cultured for 48 hours to be collected. A small amount of 1 × binding buffer was added to adjust cell density with 10 × 10^5^. After labeling, 100 ml cell suspension was added to each tube, and 5 ml Annexin V-FITC and 5 ml PI staining solution were added to each tube and then detected by a flow cytometer. The results were analyzed by Flowjo software.

### 2.6. Expression of BK_Ca_-*α*, *β*, Col IV, and FN Protein in HBZY-1Cells

Proteins extracted from the HBZY-1 cells were analyzed by Western blotting. Equal amounts of protein (about 50 *μ*g) were subjected to SDS-PAGE and transferred to a PVDF membrane, then blocked in 5% skimmed milk for 2 hours at room temperature, and then incubated overnight at 4°C with the following primary antibodies according to the instructions: anti-BK_Ca_-*α*, anti-BK_Ca_-*β*, anti-Col IV, anti-FN, and anti-*β*-actin (Abcam Biotechnology, USA). The membranes were then incubated with HRP-conjugated secondary anti-mouse antibody (Abcam Biotechnology, USA). Protein bands on the membrane were visualized by ECL (electrochemiluminescence) (ThermoScientific, Rockford, IL, USA) and quantitated using QuantityOne software (Bio-Rad, Richmond, CA, USA). Image J software was used to open the strip picture to get the gray statistics of the selected area.

### 2.7. Detection of TGF-*β*1 and Smad2/3 in Cell Supernatant by ELISA

HBZY-1 cells were inoculated into 6-well plates at 12 × 10^4^ density at 37°C in a 5% CO_2_ incubator for 24 hours and then cultured for 48 hours after intervention by grouping administration. Cell culture medium was absorbed and centrifuged at 4°C and 8000 rpm/min for 15 minutes. Only the supernatant was taken and operated according to ELISA guidelines.

### 2.8. Statistical Analysis

The experimental data were analyzed with SPSS 19.0 software (IBM, Armonk, NY, USA). The data were expressed as means ± standard deviations. *T*-test analysis was used for comparisons between two groups. One-way ANOVA was used for comparisons among multiple groups. *P* < 0.05 indicated that the difference was statistically significant.

## 3. Results

### 3.1. The Results of BK_Ca_-siRNA Transfection and NS11021 and Tet Pre-Experimental Concentration Selection

BK_Ca_-siRNA was successful transfection (seen Supplementary Figure 1), and finally BK_Ca_-*α*-1188 was selected to continue the subsequent experiment for the gene inhibition group (Supplementary Figure 2). In order to select the optimum concentration of NS11021 and Tet, a series of experiments were conducted to confirm the concentrations of NS11021 and Tet as 10 *μ*M in this study. The detailed results are given in Supplementary Figure 3.

### 3.2. Inhibition of BK_Ca_ and TGF-*β*1 Can Inhibit the Proliferation of Glomerular Mesangial Cells

**Figure 1:**
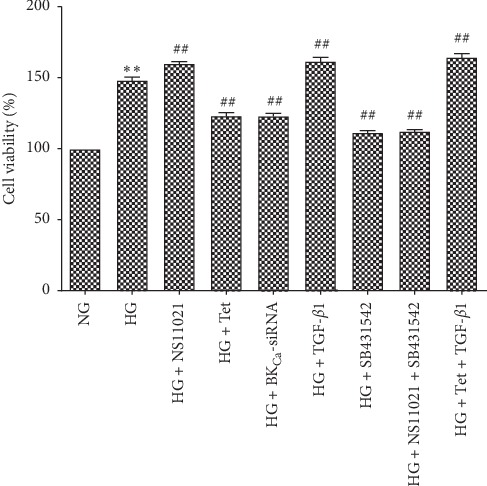
The effect of different interventions on cell viability. ^*∗*^*P* < 0.05, ^*∗∗*^*P* < 0.01 vs. NG group; ^#^*P* < 0.05, ^##^*P* < 0.01 vs. HG group.

Compared with the NG group, cell proliferation in the HG group increased significantly (*P* < 0.01). Compared with the HG group, NS11021 promoted cell proliferation (*P* < 0.01), Tet and BK_Ca_-siRNA inhibited cell proliferation (*P* < 0.01), TGF-*β*1 promoted cell proliferation (*P* < 0.01), SB431542 inhibited cell proliferation (*P* < 0.01), Tet + TGF-*β*1 promoted cell proliferation (*P* < 0.01), and NS11021 + SB431542 inhibited cell proliferation (*P* < 0.01) (Figure 1).

### 3.3. Inhibition of BK_Ca_ and TGF-*β*1 Can Suppress the Migration of Mesangial Cells

**Figure 2:**
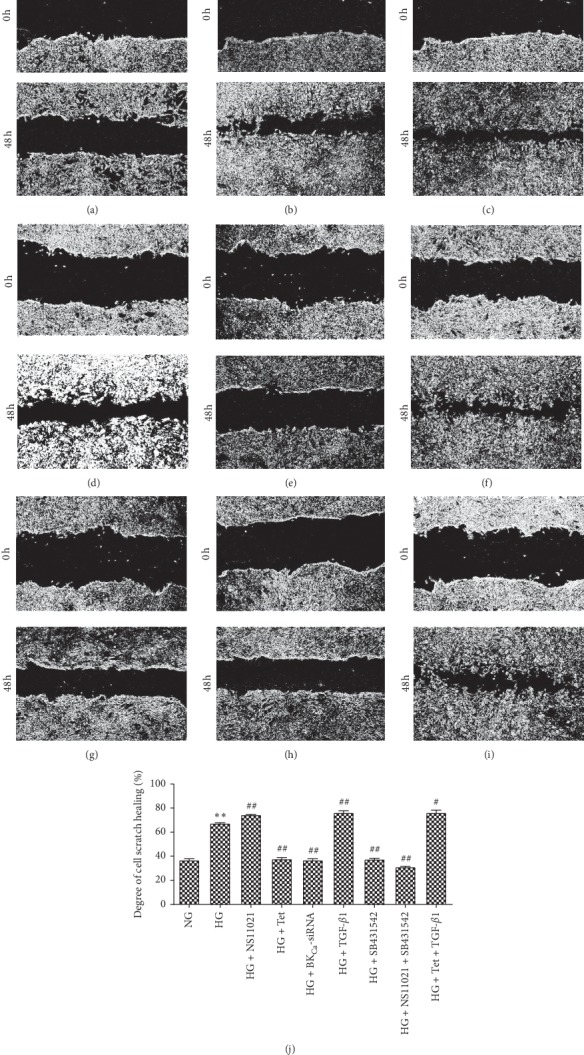
The effect of different interventions on cell migration ability was observed by an inverted microscope (×100). (a) NG group; (b) HG group; (c) HG + NS11021 group; (d) HG + Tet group; (e) HG + BK_Ca_-siRNA group; (f) HG + TGF-*β*1 group; (g) HG + SB431542 group; (h) HG + NS11021 + SB431542 group; (i) HG + Tet + TGF-*β*1 group; (j) degree of cell scratch healing in different intervention groups. ^*∗*^*P* < 0.05, ^*∗∗*^*P* < 0.01 vs. NG group; ^#^*P* < 0.05, ^##^*P* < 0.01 vs. HG group.

Compared with the NG group, the healing rate of scratches in the HG group increased from 36% to 68% after 48 hours of intervention (*P* < 0.01), indicating that the transverse migration ability of cells increased (Figures 2(a), 2(b), and 2(j)). Compared with the HG group, NS11021 enhanced cell migration (75%, *P* < 0.01) (Figures 2(c) and 2(j)), Tet decreased cell migration (39%, *P* < 0.01) (Figures 2(d) and 2(j)), BK_Ca-_siRNA decreased cell migration (38%, *P* < 0.01) (Figures 2(e) and 2(j)), TGF-*β*1 enhanced cell migration (77%, *P* < 0.01) (Figures 2(f) and 2(j)), SB431542 decreased cell migration ability (37%, *P* < 0.01) (Figures 2(g) and 2(j)), NS11021 + SB431542 decreased cell migration ability (32%, *P* < 0.01) (Figures 2(h) and 2(j)), and Tet + TGF-*β*1 enhanced cell migration ability (78%, *P* < 0.05) (Figures 2(i) and 2(j)).

### 3.4. Inhibition of BK_Ca_ Can Reduce the Apoptosis of Mesangial Cells

**Figure 3:**
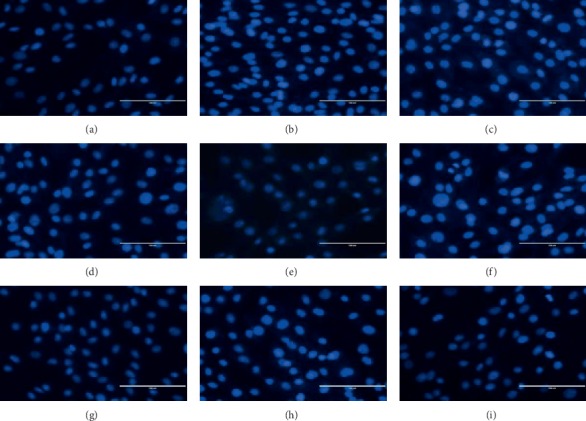
Apoptosis of each intervention group after Hoechst staining was observed by a fluorescence inversion microscope (×400). (a) NG group; (b) HG group; (c) HG + NS11021 group; (d) HG + Tet group; (e) HG + BK_Ca_-siRNA group; (f) HG + TGF-*β*1 group; (g) HG + SB431542 group; (h) HG + NS11021 + SB431542 group; (i) HG + Tet + TGF-*β*1 group. *n* = 6.

In the Hoechst staining experiment, the fluorescence intensity of the NG group, Tet group, BK_Ca_-siRNA group, SB431542 group, and NS11021 + SB431542 group was lower, most of the cell nucleus and cytoplasm were light blue, and the fluorescence expression of chromatin was uniform. The fluorescence intensity of the NG group was the lowest, and the number of apoptotic cells was the lowest (Figures 3(a), 3(d), 3(e), 3(g), and 3(h)). The fluorescence intensities of the HG group, NS11021 group, TGF-*β*1 group, and Tet + TGF-*β*1 group were high, some cell nucleus were concentrated and fragmented, and the whole staining fluorescence showed blue-white dense staining (Figures 3(b), 3(c), 3(f), and 3(i)). These results suggest that inhibiting BK_Ca_ can reduce the apoptosis of glomerular mesangial cells.

**Figure 4:**
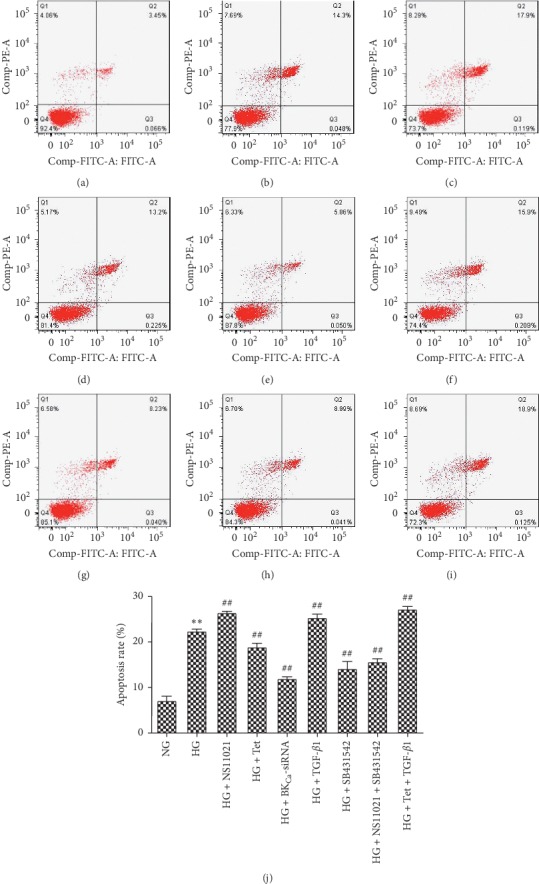
The effects of interventions on apoptosis were examined by flow cytometry. (a) NG group; (b) HG group; (c) HG + NS11021 group; (d) HG + Tet group; (e) HG + BK_Ca_-siRNA group; (f) HG + TGF-*β*1 group; (g) HG + SB431542 group; (h) HG + NS11021 + SB431542 group; (i) HG + Tet + TGF-*β*1 group; (j) apoptosis rate in different intervention groups. ^*∗*^*P* < 0.05, ^*∗∗*^*P* < 0.01 vs. NG group; ^#^*P* < 0.05, ^##^*P* < 0.01 vs. HG group. *n* = 6.

Flow cytometry was used to further detect the apoptotic status of each intervention group. Compared with the NG group, the apoptotic rate of the HG group increased from 7.6% to 22.1% after 48 hours of intervention (*P* < 0.01), indicating that apoptotic rate increased (Figures 4(a), 4(b), and 4(j)). Compared with the HG group, the apoptotic rate of NS11021 cells increased (26.3%, *P* < 0.01) (Figures 4(c) and 4(j)). Tet cells decreased (18.6%, *P* < 0.01) (Figures 4(d) and 4(j)), and BK_Ca_-siRNA cells decreased (12.2%, *P* < 0.01) (Figures 4(e) and 4(j)). The apoptotic rate of TGF-*β*1 cells increased (25.6%, *P* < 0.01) (Figures 4(f) and 4(j)), SB4315). The apoptotic rate of 42 cells decreased (14.9%, *P* < 0.01) (Figures 4(g) and 4(j)). The apoptotic rate of NS11021 + SB431542 cells decreased (15.7%, *P* < 0.01) (Figures 4(h) and 4(j)). The apoptotic rate of Tet + TGF-*β*1 cells increased (27.7%, *P* < 0.01) (Figure 4(i)).

### 3.5. Inhibition of BK_Ca_ Can Inhibit the Expression of Col IV and FN Protein in Glomerular Mesangial Cells

**Figure 5:**
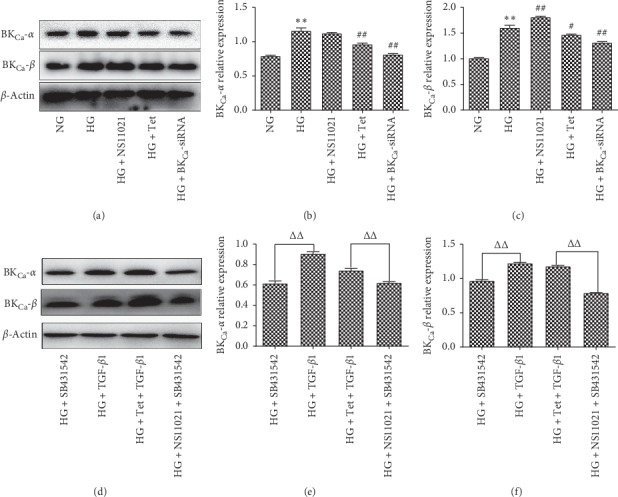
The effects of each group on the expression of BKCa-*α* and *β* protein were detected by Western blotting. ^*∗*^*P* < 0.05, ^*∗∗*^*P* < 0.01 vs. NG group; ^#^*P* < 0.05, ^##^*P* < 0.01 vs. HG group; ^△△^*P* < 0.01. *n* = 6.

Compared with the NG group, the expression of BK_Ca_-*α* and *β* protein in the HG group increased 48 hours after intervention (*P* < 0.01). Compared with the HG group, the expression of BK_Ca_-*α* in NS11021 cells was insignificant (*P* < 0.05), while the expression of BK_Ca_-*β* in NS11021 cells increased (*P* < 0.01). Tet decreased the expression of BK_Ca_-*α* and *β* in NS11021 cells (*P* < 0.05), and BK_Ca_-siRNA decreased the expression of BK_Ca_-*α* and *β* in NS11021 cells (*P* < 0.01) (Figures 5(a)–5(c)). Compared with SB431542, NS11021 + SB431542, and Tet + TGF-*β*1, the expression of BK_Ca_-*α* and *β* protein in cells was significantly different (*P* < 0.01) (Figures 5(d)–5(f)).

**Figure 6:**
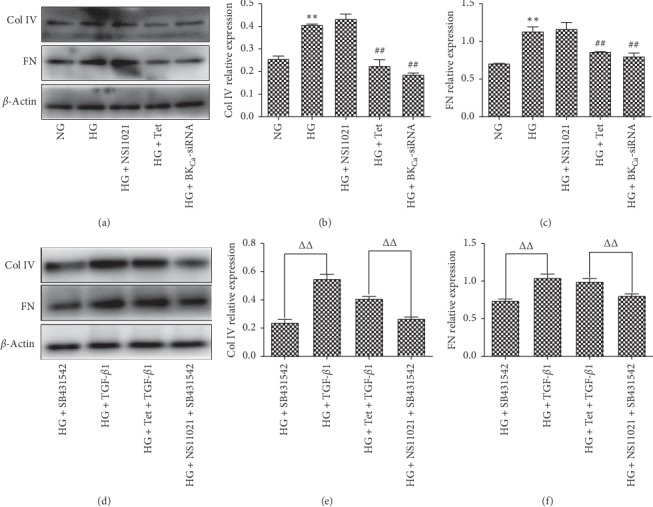
The effects of each group on the expression of collagen IV and fibronectin were detected by Western blotting. ^*∗*^*P* < 0.05, ^*∗∗*^*P* < 0.01 vs. NG group; ^#^*P* < 0.05, ^##^*P* < 0.01 vs. HG group; ^△△^*P* < 0.01.*n* = 6.

Compared with the NG group, the expression of Col IV and FN in the HG group increased 48 hours after intervention (*P* < 0.01). Compared with the HG group, there was no significant difference in the expression of Col IV and FN in NS11021 cells (*P* < 0.05), Tet decreased the expression of Col IV and FN in NS11021 cells (*P* < 0.01), and BK_Ca_-siRNA decreased the expression of Col IV and FN in NS11021 cells (*P* < 0.01) (Figures 6(a)–6(c)). Compared with SB431542, NS11021 + SB431542, and Tet + TGF -*β*1, the expression of Col IV and FN in cells was significantly different (*P* < 0.01) (Figures 6(d)–6(f)).

### 3.6. Inhibition of BK_Ca_ Can Inhibit the Expression of TGF-*β*1 and Smad2/3 in Supernatant of Mesangial Cells

**Figure 7:**
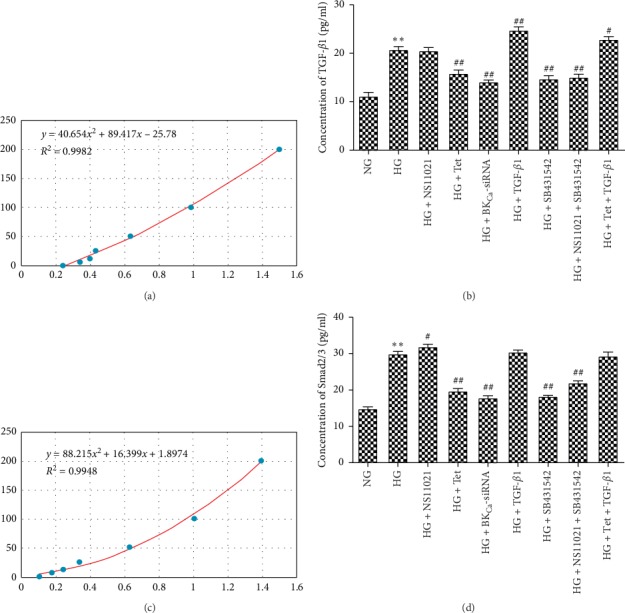
The expression of TGF-*β*1 and Smad2/3 in each intervention group. (a) Quantitative map of TGF-*β*1 concentration in each group; (b) quantitative map of Smad 2/3 concentration in each group ^*∗*^*P* < 0.05, ^*∗∗*^*P* < 0.01 vs. NG group;^#^*P* < 0.05, ^##^*P* < 0.01 vs. HG group.

Compared with the NG group, the expression of TGF-*β*1, and Smad2/3 increased in the HG group (*P* < 0.01). Compared with the HG group, the expression of Smad2/3 increased in the NS11021 group (*P* < 0.05), but the expression of TGF-*β*1 did not change significantly (*P* < 0.05); the expression of TGF-*β*1 and Smad2/3 decreased in the Tet group, BK_Ca-_siRNA group, SB431542 group, and NS11021 + SB431542 group (*P* < 0.01); the expression of TGF-*β*1 increased significantly in the TGF-*β*1 group (*P* < 0.01), while the expression of Smad2/3 did not change significantly (*P* < 0.05). The expression of Smad2/3 increased (*P* < 0.05), but the difference of Smad2/3 expression was insignificant (*P* < 0.05) (Figures 7(a) and 7(b)).

## 4. Discussion

DKD is a common complication of DM, which is one of the three diseases with the highest incidence at present. Current research shows that the core of DKD is glomeruli [5]. MCs are the most active intrinsic cells in the glomerulus. They can be regulated by some cytokines to abnormally grow and secrete extracellular matrix when the glomerulus is damaged. They are important pathogenic factors leading to glomerulosclerosis [22]. As the most active intrinsic cell in the glomerulus, MCs can hypertrophy and proliferate in the early stage of DKD and further induce multidirectional signal pathways to affect renal function. The glomerular mesangial dilatation not only induces the secretion and deposition of macromolecule substances such as ECM but also squeezes the glomerular capillaries to make them narrow or even blocked and promotes the release of inflammatory mediators such as DKD-related cytokines and growth factors [5, 18–23]. Glomerular lesions of DKD include proliferation and hypertrophy of MCs, excessive accumulation of ECM in the form of abnormally increased mesangial matrix, and thickening of glomerular basement membrane, which eventually leads to nodular glomerulosclerosis [24, 25]. Thus, abnormal proliferation, migration, apoptosis of MCs, and abnormal accumulation of ECM play a key role in the process of glomerular lesion. The results of cell proliferation, scratch, and apoptosis in this study showed that NS11021 could significantly promote the proliferation, migration, and apoptosis of HBZY-1 cells compared with the HG group. On the contrary, Tet and BK_Ca_-siRNA could significantly inhibit the proliferation, migration, and apoptosis of HBZY-1 cells. These results suggest that the proliferation, migration, and apoptosis of HBZY-1 cells can be inhibited by inhibiting BK_Ca_, thus improving glomerular lesions. It should be noted that the cell proliferation and apoptotic rate in the HG group increased at the same time, which is different from that in most experiments. It suggests that high glucose has a two-way effect of nutrition and damage to cells and can promote the proliferation and apoptosis of HBZY-1 cells at the same time. Compared with the HG group, NS11021 did not promote the secretion of Col IV and FN in HBZY-1 cells. The specific reason needs further study. It is suspected that the increasing open rate of BK_Ca_ in high-glucose environment leads to the weak effect of agonists. However, Tet and BK_Ca_-siRNA can significantly inhibit the secretion of Col IV and FN in HBZY-1 cells. It is suggested that the secretion of Col IV and FN in ECM of HBZY-1 cells can be inhibited by inhibiting BK_Ca_ to improve glomerular lesions in another way.

TGF-*β* is one of the central factors in the occurrence and development of DKD. It can induce the proliferation of mesangial cells and the secretion of collagen and even directly induce the synthesis of ECM such as Col and FN from transcriptional level to promote the process of renal fibrosis [26]. At present, three subtypes of TGF-*β*1, 2, and 3 have been identified in mammals [27], among which TGF-*β*1 is most closely related to kidney [28]. Smad2 and 3, as the main downstream effector proteins of TGF-*β* signaling pathway, can bind to Smad4 after phosphorylation and then transfect into nuclear regulatory gene transcription. Among them, Smad3 plays a more important role and is recognized as a fibrogenic factor [29].

## 5. Conclusion

TGF-*β*1 has similar effects to NS11021, which can promote the proliferation, migration, and apoptosis of HBZY-1 cells and promote the secretion of Col IV and FN in ECM. And SB431542 is similar to Tet and BK_Ca_-siRNA, which suggests that BK_Ca_ may be related to TGF-*β*1/Smad2/3 pathway. Further experiments showed that NS11021 + SB431542 could inhibit the proliferation, migration, and apoptosis of HBZY-1 cells and the secretion of Col IV and FN in ECM. On the contrary, Tet + TGF-*β*1 could promote the proliferation, migration, and apoptosis of HBZY-1 cells and the secretion of Col IV and FN in ECM, suggesting that TGF-*β*1/Smad2/3 and BK_Ca_ regulate the proliferation, migration, and apoptosis of HBZY-1 cells and the secretion of Col IV and FN are the same signaling pathway. TGF-*β*1/Smad2/3 is downstream of BK_Ca_. Through the development of specific agonists or blockers of BK_Ca_ to regulate the expression and function of BK_Ca_, it may provide a new therapeutic direction for DKD.

## Data Availability

The datasets used and/or analyzed in the study are available from the corresponding author on reasonable request.

## Supplementary Materials

Supplementary Materials Supplementary Figures 1 and 2: the results of siRNA interference. Supplementary Figure 3: detailed results of different concentrations of NS11021 and Tet to treat cells.
