# Supplementary material for: BKCa Mediates Dysfunction in High Glucose Induced Mesangial Cell Injury via TGF-β1/Smad2/3 Signaling Pathways
Source: Int J Endocrinol. 2020 Apr 29;2020:3260728. doi: 10.1155/2020/3260728 (PMC7206888; doi:10.1155/2020/3260728)
Supplement: Supplementary Materials — Supplementary Figures 1 and 2: the results of siRNA interference. Supplementary Figure 3: detailed results of different concentrations of NS11021 and Tet to treat cells. [file 3260728.f1.docx]

Supplementary Materials

**RNA interference**

HBZY-1 cells were transfected with either different siRNA targeting BKCa, β-actin, or negative control siRNA (purchased from sigma, China ) according to the manufacturer’s instructions. The targeting siRNA sequence for BKCa -α-749, BKCa -α-1188 and BKCa-α-1501 are 5’-GCAUGUGGUGGGCUUUCUUTT-3’ , 5’-GCGGUUUAUUGCAGCCAAUTT-3’and 5’-GCACUUACGUACUGGGAAUTT-3’, respectively. The siRNA sequence for β-actin, negative control and Fluorescent Contro l(FAM) group is 5’-CUCUGAACCCUAAGGCCAATT-3’, 5’-UUCUCCGAACGUGUCACGUTT-3’ and 5’-UUCUCCGAACGUGUCACGUTT-3’, respectively. After transfection, the cells were cultured for 6h to change medium and photo the picture. And then the cells were cultured for 48 h to detect mRNA by the method of qPCR.

**A preliminary study conducted on the concentration of Tet and NS11021**

In order to further select the Tet and NS11021 concentrations, hbzy-1 cells were exposed to Tet and NS11021 of different concentrations the concentration gradients were 0.1, 1, 10, 100uM and 0.1, 1, 10, 100uM ,respectively. Cell viability was determined by incubation for 24h and 48h.

**Results**

**BKCa-siRNA was successfully transfected**

Hbzy-1 cells in the FAM group were observed at 480nm wavelength under an inverted fluorescence microscope at 6h after transfection. Green fluorescence was seen scattered in the cytoplasm under the field of view, indicating that siRNA was transfected into the cells and the operation procedure was successful (seen fig s1) .


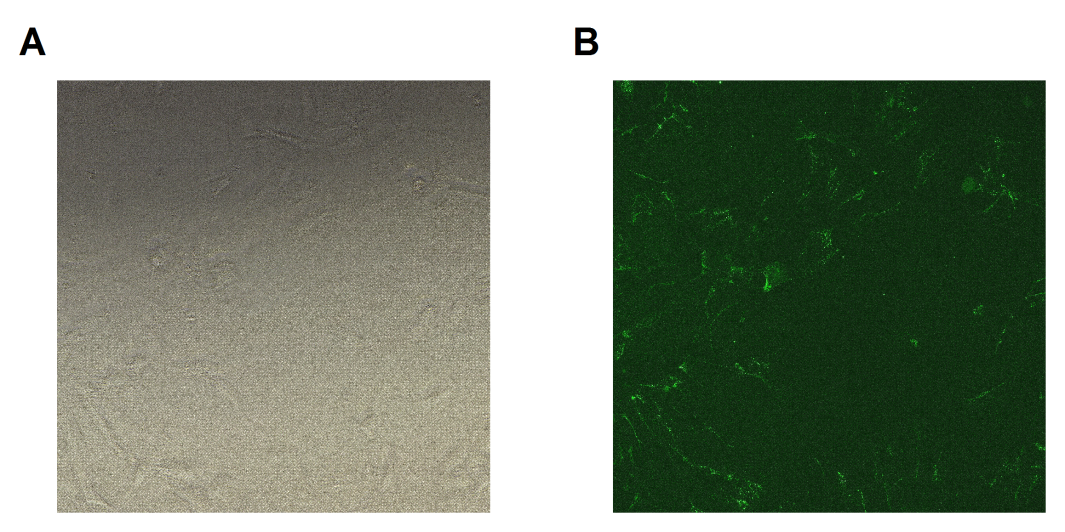


Figure s1. Detection of transfection efficiency in the FAM group by fluorescence inversion microscopy（×400）. A. white light; B. 480 nm fluorescence.

The mRNA of BKCa-α was detected by qPCR after transfection 48h of 3 siRNA target genes, and the expression was calculated by using the amplification formula 2^-△△Ct^ analysis. The dissolution curves of BKCa-α and β-actin were smooth (fig. s2.a), with a single dissolution peak (fig. s2.b).The dissolution temperature of BKCa –siRNA and β-actin were 84.5℃ and 77℃, respectively. (fig s2.a), indicating high specificity of primer. The analysis of the data showed that the mRNA expression of BKCa-α-1188 was significantly lower than that of BKCa-α-749 and BKCa-α-1501 (P<0.05) (fig s2.c), while the difference between BKCa-α-749 and BKCa-α-1501 was not statistically significant (fig s2.d).Therefore, BKCa-α-1188 was selected to continue the subsequent experiment for gene inhibition group.


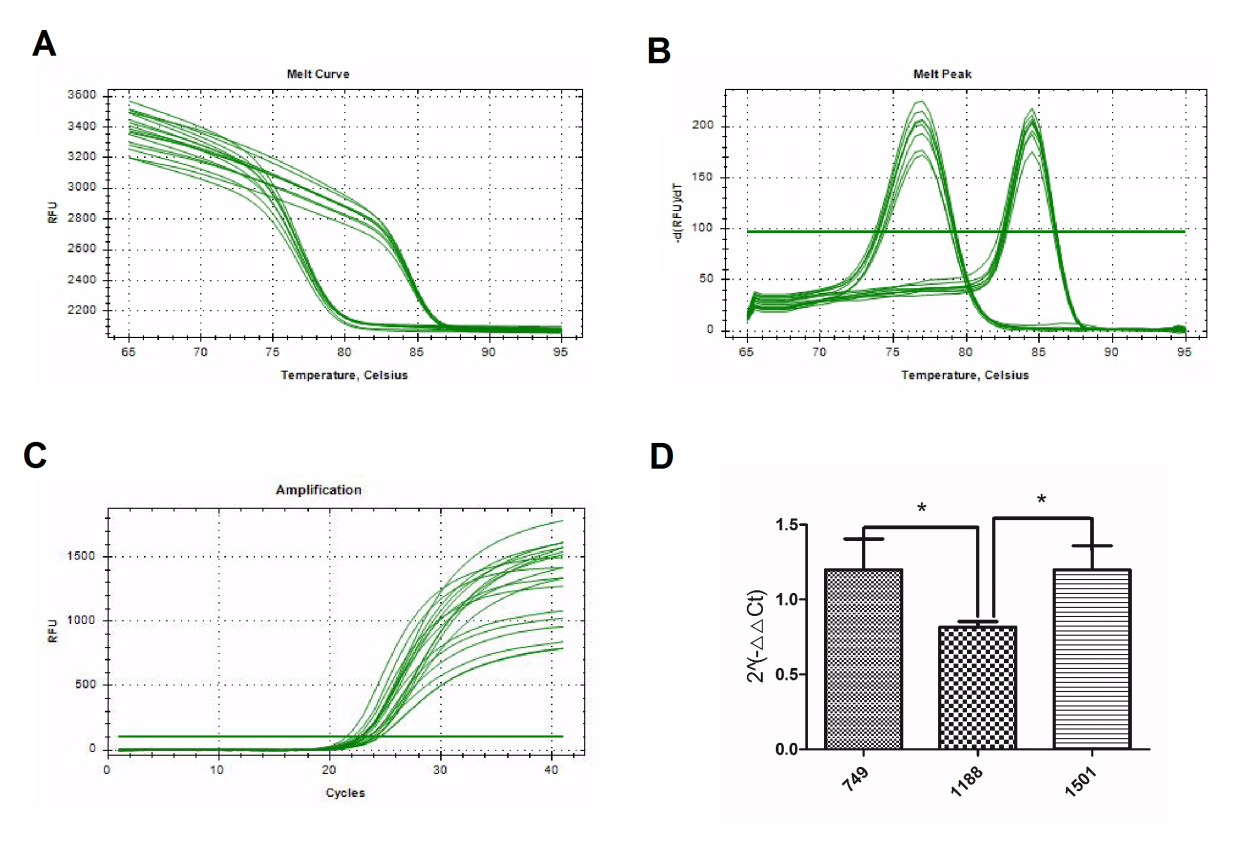


Figure s2. BK_Ca_-α-siRNA amplification reaction. A. melting curve; B. melting peak; C. amplification curve; D. BK_Ca_-α-siRNA749, BK_Ca_-α-siRNA1188, BK_Ca_-α-siRNA1501 relative expression amount(^*^*P*＜0.05).

**The results of NS11021 and Tet pre-experimental concentration selection**

Compared with NG group, cell viability of HG group increased significantly at 24h and 48h (P < 0.01).Compared with HG group, cell viability of NS11021-100μm group and Tet -10μm group decreased significantly at 24h and 48h (P < 0.01), cell viability of NS11021-10μm group increased at 24h and 48h (P < 0.05), cell viability of NS11021-1μm group, NS11021-0.1μm group, Tet -1μm group and Tet -0.1μm group did not change significantly at 24h and 48h (P > 0.05).However, compared with NG group, NS11021-100μm group and Tet -100μm group had lower cell viability at 24h and 48h (P < 0.01), suggesting that the drug concentration was too high to cause cell death. (fig.s3). NS11021 and Tet 100μM were both lower in cell viability than NG group and had a greater effect on cell damage, so they were excluded. The effects of 1μM and 0.1μm were not different from those of HG group. Therefore, NS11021 and Tet with both 10μM concentrations were selected in this study to continue the experiment.


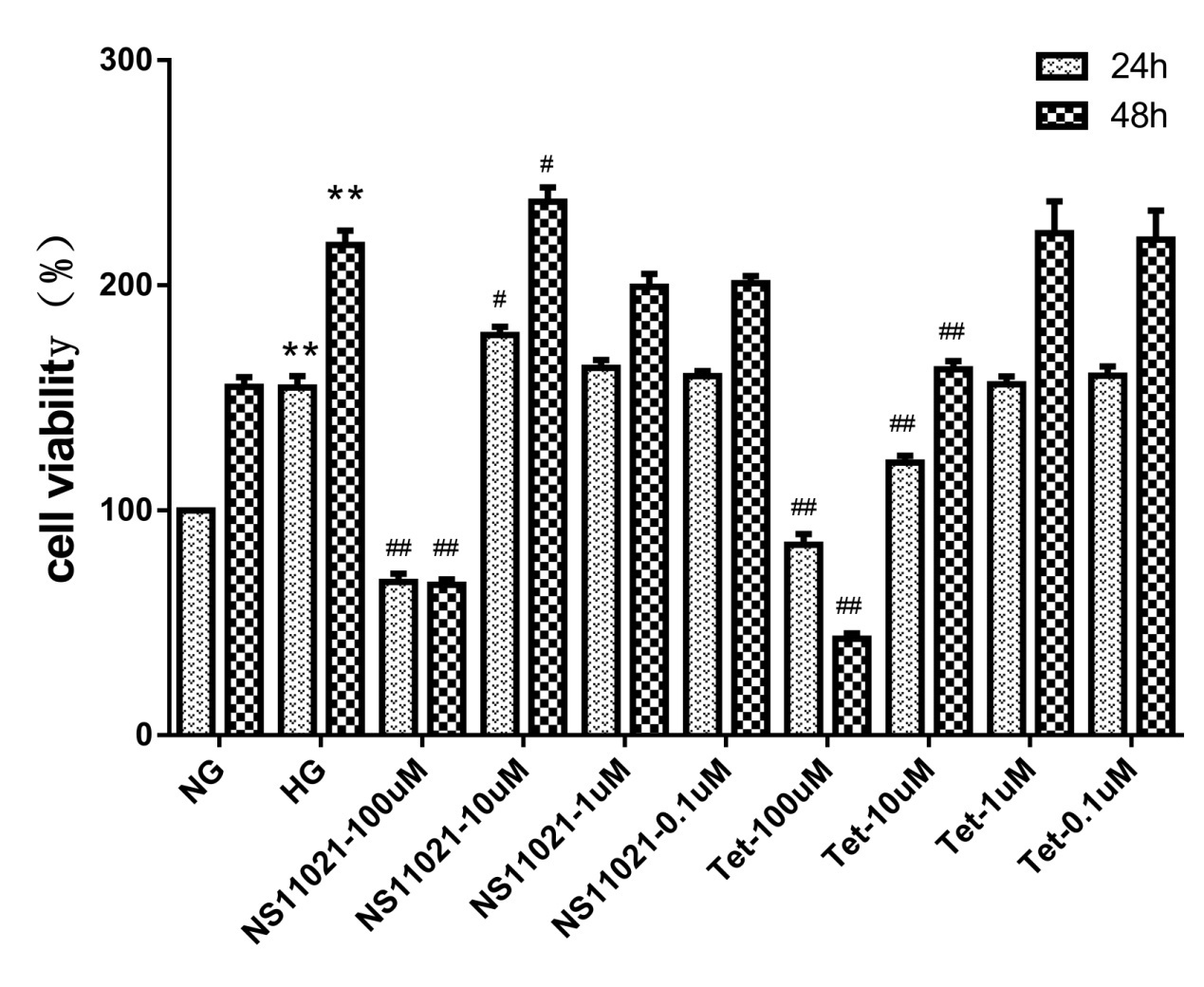


Figure s3. Effects of different concentrations of NS11021 and Tet on cell viability. (Compared with NG group ^*^*P*＜0.05, ^**^*P*＜0.01; compared with HG group ^#^*P*＜0.05, ^##^*P*＜0.01)
